# Collargolum in Gonorrhea

**Published:** 1905-08

**Authors:** 


### Collargolum in Gonorrhea.

Since publishing a note on the use of collargolum for urethro-vesical lavage, Dr. Tansard, of Prof. Balzer's service at Hospital Saint Louis, Paris, has successfully employed it in numerous cases. Thus, by means of two daily autoirrigations with a quart of a 1.500 collargolum solution a urethritis that had persisted for several months was cured in 10 days. There was never the least irritation.

In old and chronic urethritis with profound lesions of the mucosa and periurethral cysts, the following method gave excellent results: massage on a Béniqué 55 or 60 sound; urethral lavage with antiseptic solution; and finally a permanent injection of 30 to 45 minims of a 4 per cent. collargolum solution, the urethra being closed with cotton. This did especially good service in a case of over two years' standing which resisted all other treatment. Four cases were all cured within 18 days. In three others he alternated, with good results, collargolum irrigations with oxycyanide of mercury lavage.

Tansard further treated acute gonorrheas with oxycyanide of mercury until the discharge had nearly dried up, and then gave collargolum instillation, the filament disappearing from the urine after 5 to 8 of them. Ten cases of chronic urethritis,—some more than 3 years old,—did not remain under observation until cured; but in all the gonococcus disappeared after less than 30 instillations, though there were still filaments in the morning urine.

In six gonorrheal cystites about 1 dram of a 4 per cent. solution was injected daily into the bladder. In five every vesical symptom disappeared within 8 days; the sixth, which was very violent, remained unimproved after 10 days and was put on silver nitrate, which acted no better.

Tansard concludes that collargolum is an important antigonorrheic, both in acute and chronic cases. It acts as rapidly as the silver salts. Urethra and bladder show a remarkable tolerance for it; the patient suffers no pain and has no desire to urinate after its use. Collargolum is not at all caustic; it is the silver itself that combats and destroys the gonococcus in the urethra and bladder.—*Journal des Praticiens,* May 20, 1905.

